# ISSUES OF LIFE-SUSTAINING TREATMENT DECISION ACT IN CLINICAL PRACTICE FOR INDIVIDUALS AT THE END OF LIFE

**DOI:** 10.1093/geroni/igad104.2922

**Published:** 2023-12-21

**Authors:** Eunjeong Song, Dongsoon Shin, Jooseon Lee, Minjeong Eom, Suhee Oh, Seon Young Yun, Heejung Lee, Rhayun Song

**Affiliations:** Chungnam National University Hospital, Daejeon, Republic of Korea; Chungnam National University Hospital, Daejeon, Republic of Korea; Chungnam National University Hospital, Daejeon, Republic of Korea; Chungnam National University Hospital, Daejeon, Republic of Korea; Chungnam National University Hospital, Daejeon, Republic of Korea; Chungnam National University Hospital, Daejeon, Republic of Korea; Chungnam National University Hospital, Daejeon, Republic of Korea; Chungnam National University Hospital, Daejeon, Republic of Korea

## Abstract

This study aimed to investigate the status and issues related to the patient’s decision-making regarding life-sustaining treatment (LST) in clinical practice and to provide strategic suggestions to improve the hospital policy. The retrospective observational study was conducted with adult patients, admitted between Jan 1 and Dec 31, 2021, who went through the end-of-life process and died in a university hospital in Korea. The electronic data included age, gender, admission date, expiration date, and Do-not-attempt-resuscitation (DNAR) and LST-related data (date to sign, who signed, and clinical state when signed). A total of 916 patients were included. Only 4.1% of the patients decided on LST by themselves, while the decision was made mainly by the family, specifically children (54.2%). The time of signing DNAR consent was before eight days from the date of death (33.7%) and the previous day of the death (27.6%). While early warning score was used to predict the critical state of the patients, the period from the date of sign to the date of death was 8.9 (SD=25.7) days for DNAR and 2.76 (SD=6.95) days for LST. Strategic suggestions were made to facilitate the timing of signing the consent form for LST based on the early warning score, and encourage patient involvement in the decision-making process. Further efforts are required to improve the awareness of patients and medical staff on the implementation of LST and to clarify the ambiguity of the timing of document preparation.

